# Implication of microRNA-140 in osteoarthritis

**DOI:** 10.1186/ar3580

**Published:** 2012-02-09

**Authors:** Johanne Martel-Pelletier

**Affiliations:** 1 University of Montreal and Osteoarthritis Research Unit, Notre-Dame Hospital, CRCHUM, Montreal, Quebec, Canada

## 

In osteoarthritis (OA), despite major progress regarding the identification and roles of catabolic mediators, further knowledge about factors regulating their expression is needed. In this line of thought, one recently identified class of molecules, the microRNA (miRNA), has been found to add another level of regulation to gene expression by down-regulating its target genes. miRNAs are 20-23 nucleotides (nt)-long single-stranded non-coding RNA molecules that act as transcriptional repressors by binding to the 3' untranslated region (UTR) of the target messenger RNA. Recently, miR-140 has emerged as being implicated in OA by modulating genes involved in the pathogenesis of this disease. The miRNA-140 gene is located between exons 16 and 17 in one intron of the WW domain containing the E3 ubiquitin protein ligase 2 (WWP2) gene [1]. The miR-140, originally found in cartilage [2], has recently been linked more specifically to the OA process [3,4]. The miRNA-140 decreases the expression of some genes known to play detrimental roles in OA cartilage. Those genes include histone deacetylase 4 (HDAC4) [2,5], ADAMTS-5 [6,7], Smad3 [8,9], and IGFBP5 [3]. On human chondrocytes, the expression level of miR-140 was found to be significantly decreased in OA compared to normal [3,4], thus favouring an increased expression of its target genes and consequently a role in OA progression. Interestingly, further investigation of the transcriptional regulation of miR-140 showed that in human OA chondrocytes miR-140 also has a WWP2-independent regulation. This occurs through the miR-140 intronic regulatory sequence in which the transcription factor NFAT3 acts directly and NFAT5 indirectly through the growth factor TGF-β1/Smad3. These data are of importance as they can provide a new basis for the rationalization of a therapeutic strategy for this disease.
